# The CRISPR-associated Cas4 protein Pcal_0546 from *Pyrobaculum calidifontis* contains a [2Fe-2S] cluster: crystal structure and nuclease activity

**DOI:** 10.1093/nar/gku797

**Published:** 2014-09-08

**Authors:** Sofia Lemak, Boguslaw Nocek, Natalia Beloglazova, Tatiana Skarina, Robert Flick, Greg Brown, Andrzej Joachimiak, Alexei Savchenko, Alexander F. Yakunin

**Affiliations:** 1 Department of Chemical Engineering and Applied Chemistry, University of Toronto, Toronto, Ontario M5S 3E5, Canada; 2 Midwest Center for Structural Genomics and Structural Biology Center, Biosciences Division, Argonne National Laboratory, Argonne, IL 60439, USA

## Abstract

Cas4 nucleases constitute a core family of CRISPR (Clustered Regularly Interspaced Short Palindromic Repeats) associated proteins, but little is known about their structure and activity. Here we report the crystal structure of the Cas4 protein Pcal_0546 from *Pyrobaculum calidifontis*, which revealed a monomeric protein with a RecB-like fold and one [2Fe-2S] cluster coordinated by four conserved Cys residues. Pcal_0546 exhibits metal-dependent 5′ to 3′ exonuclease activity against ssDNA substrates, whereas the Cas4 protein SSO1391 from *Sulfolobus solfataricus* can cleave ssDNA in both the 5′ to 3′ and 3′ to 5′ directions. The active site of Pcal_0546 contains a bound metal ion coordinated by the side chains of Asp123, Glu136, His146, and the main chain carbonyl of Ile137. Site-directed mutagenesis of Pcal_0546 and SSO1391 revealed that the residues of RecB motifs II, III and QhXXY are critical for nuclease activity, whereas mutations of the conserved Cys residues resulted in a loss of the iron-sulfur cluster, but had no effect on DNA cleavage. Our results revealed the biochemical diversity of Cas4 nucleases, which can have different oligomeric states, contain [4Fe-4S] or [2Fe-2S] clusters, and cleave single stranded DNA in different directions producing single-stranded DNA overhangs, which are potential intermediates for the synthesis of new CRISPR spacers.

## INTRODUCTION

Clustered Regularly Interspaced Short Palindromic Repeats (CRISPR) and associated proteins (Cas) represent the only adaptive microbial immunity system which protects microbial cells from invading viruses and plasmids (1,2). This immunity system is based on the incorporation of short DNA sequences (30–50 nucleotides) from viral genomes or plasmids into the host chromosome, which are then transcribed into guide RNAs (CRISPR RNAs or crRNAs) and direct Cas proteins to specifically degrade DNAs or RNAs containing the complementary sequences (3–6). CRISPR-based immunity includes three steps: adaptation, expression and interference. During the adaptation step, Cas proteins recognize foreign DNA and incorporate short DNA fragments (30–50 nucleotides) as new spacers separated by identical repeats of similar size into the CRISPR locus on the host chromosome. These viral-specific spacers are transcribed during the expression step as long primary crRNAs and processed by other Cas proteins into a library of short crRNAs containing one spacer flanked by portions of the adjacent repeat sequences. In the CRISPR interference step, these short crRNAs guide different Cas proteins or complexes to specifically recognize and degrade foreign DNAs or RNAs containing complementary sequences.

At least 65 different Cas proteins are encoded by gene clusters located close to CRISPR loci (1,7,8). Six Cas families (Cas1 to Cas6) comprise the core cluster of CRISPR-associated proteins with Cas1 and Cas2 proteins found in all CRISPR-containing organisms. Based on the composition of the Cas operons and phylogeny, the CRISPR systems have been classified into three types: I, II and III. In addition to Cas1 and Cas2 proteins, the major CRISPR system I includes the helicase/nuclease Cas3 and the Cascade complex of 4–5 Cas proteins involved in the processing of the crRNAs and recognition of DNA targets (5,8,9). This system is the most widespread and is divided into six subtypes (I-A to I-F) depending on the presence of subtype-specific genes. CRISPR types II and III have no Cas3 proteins, but include the large multidomain protein Cas9 (type II) or Cas6+RAMP module (type III) (10–12).

The core Cas4 proteins are present in CRISPR systems I-A, I-B, I-C, I-D and II-B and have been predicted to be involved in the adaptation step, because their genes are often associated with Cas1 genes (13). In addition, in some organisms (e.g. *Leptospira*, *Rhodospirillum* and *Myxococcus*) the Cas4 protein is covalently fused to a Cas1 domain suggesting that these protein domains are functionally related (14). Based on sequence, there are two groups of Cas4 proteins: DUF83 (Cas4) and DUF911 (Csa1 or Cas4′), which have different chain lengths (171–221 aa for DUF83 and 265–362 aa for DUF911) (7,8,15). Both sub-families contain the key motifs of the RecB nuclease family (I, II, III and QhXXY) and four conserved Cys residues (one at the N-terminus and three at the C-terminus), which are predicted to coordinate an Fe-S cluster (15–18). This was confirmed by the recent crystal structure of the Cas4 protein SSO0001 from *Sulfolobus solfataricus*, which revealed the presence of one [4Fe-4S] cluster coordinated by the four conserved Cys residues in each protomer (19). The characterized RecB-like nucleases are part of the multifunctional helicase-nuclease complexes RecBCD from *Escherichia coli* and AddAB from *Bacillus subtilis* involved in DNA recombination and repair, as well as in degradation of foreign DNA entering the microbial cell (20–22).

Presently, the *S. solfataricus* protein SSO0001 represents the only biochemically and structurally characterized Cas4 protein, which exhibits metal-dependent endonuclease and 5′ → 3′ exonuclease activities against single stranded DNA (ssDNA) and ATP-independent unwinding activity toward double stranded DNA (dsDNA) (16,19). Its crystal structure has revealed a toroidal decameric complex with each protomer containing one [4Fe-4S] cluster coordinated by the four conserved Cys residues (19). These cysteines comprise a signature motif of ‘iron staple’ nucleases, which have been proposed to use iron–sulfur clusters for the stabilization of the RecB-like nuclease domain (16,23). The important role of the SSO0001 [4Fe-4S] cluster for its nuclease activity was confirmed by site directed mutagenesis of the cluster-coordinating Cys residues (19). Based on the biochemical characterization of SSO0001, it has been proposed that Cas4 proteins can have at least two functions in CRISPR-based immunity including the generation of 3′-overhangs for integration of new spacers and direct degradation of foreign DNAs (16,19).

Here we show that in contrast to SSO0001 the Cas4 protein Pcal_0546 from *Pyrobaculum calidifontis* contains one [2Fe-2S] cluster coordinated by four conserved Cys residues. Pcal_0546 has endonuclease and 5′ → 3′ exonuclease activities against ssDNA, whereas the Cas4 protein SSO1391 from *S. solfataricus* cleaves ssDNA in both directions. Alanine replacement mutagenesis revealed that the RecB motif residues are required for nuclease activity of both proteins, whereas mutations of the cluster-coordinating Cys residues disrupted the [Fe-S] cluster, but had no significant effect on DNA cleavage.

## MATERIALS AND METHODS

### Protein expression, purification and mutagenesis

Cloning and purification of the 6His-tagged SSO1391 and Pcal_0546, as well as site-directed mutagenesis were performed as described previously (24). Several clones (K138A and H146A of Pcal_0546 and C279A of SSO1391) produced no soluble proteins, and the respective mutant proteins were not purified. The oligomeric state of purified proteins was analyzed using size-exclusion chromatography on a Superdex S200 10/300 GL column (Amersham Biosciences) (19).

### Preparation of nucleic acid substrates and enzymatic assays

The ssDNA oligonucleotides used in this work (Supplementary Table S1) were purchased from IDT (USA). The oligonucleotides were [^32^P]-labeled at the 3′-end using calf thymus terminal transferase TdT or at the 5′-end using T4 polynucleotide kinase (PNK, BioLabs) and purified as previously described (24). The synthetic dsDNA or splayed arms substrates were prepared by annealing the oligonucleotides shown in Supplementary Tables S1 and S2.

Unless stated otherwise, ssDNase or unwinding assays with Pcal_0546 were carried out at 45°C in a reaction mixture containing 50 mM Tris-HCl (pH 8.0), 5 mM (for exonuclease assays) or 2 mM (for endonuclease assays) MgCl_2_, 1 mM DTT (for exonuclease assays), 0.1 μM 3′- or 5′-[^32^P]-labeled substrate or 5 nM ssDNA of the M13mp18 phage. For SSO1391, ssDNA exonuclease or unwinding assays were carried out at 45°C using a reaction mixture containing 50 mM MES (pH 6.5), 5 mM KCl, 5mM CoCl_2_ and 0.1 μM 3′- or 5′-[^32^P]-labeled substrate. Endonuclease assays were carried out at 45°C and the reaction mixture contained 50 mM Tris (pH 8.5), 50 mM KCl, 0.5 mM MnCl_2_ and 5 nM ssDNA of the M13mp18 phage, or 50 mM Tris-HCl (pH 8), 5 mM KCl, 0.05 mM MnCl_2_ and 5 nM ssDNA of the M13mp18 phage. The exonuclease reactions were analyzed using denaturing gel electrophoresis (15% polyacrylamide and 8 M urea), whereas agarose gel electrophoresis (0.9%) was used for endonuclease assays (25).

The assay for the liquid chromatography-mass spectrometry (LC-MS) analysis of the Pcal_0546 and SSO1391 reaction products was performed using optimal reaction conditions with 55 μM Pcal_0546 or 42 μM SSO1391 and poly-dC_10_ substrate (10 μg). The LC-MS analysis of the products of poly-dC_10_ cleavage was performed essentially as described before (19) using a Q-Exactive mass spectrometer with HESI source and an Ulti-Mate 3000 UHPLC/autosampler system (all from Thermo Scientific). Separation by liquid chromatography was conducted on a Hypersil Gold C18 column (50 mm × 2.1 mm, 1.9 μm particle size, Thermo Scientific) equipped with a guard column. The pump was run at a flow rate of 200 μl/min. Solvent A was water containing 0.1% formic acid; solvent B was methanol. The gradient was 100% A from 0 to 4 min; linear gradient to 100% B from 4 to 10 min; 100% B from 10 to 20 min; linear gradient to 100% A from 20 to 20.5 min; 100% A from 20.5 min to 30 min. The autosampler temperature was maintained at 4°C and the injection volume was 10 μl. Instrument settings were as follows: negative ionization mode, scan range m/z 120–800, resolution 70 000 at 1 Hz, AGC target 1e6, maximum injection time 50 ms, sheath gas flow rate 25 (arbitrary units), auxiliary gas flow rate 3 (arbitrary units), spray voltage −3.3 kV and capillary temperature 320°C.

### Crystallization and structure determination of Pcal_0546

The sitting-drop vapor-diffusion method was used to obtain crystals of Pcal_0546 at room temperature. Reddish crystals were grown by mixing 1 μl of the protein (10 mg/ml) with 1 μl of the crystallization solution containing (3.5 M sodium formate, and 100 mM Bis-Tris-Propane pH 7). Crystals were stabilized with the crystallization buffer containing 7% PEG 200, 7% sucrose and 7% glycerol followed by cryoprotection in Paratone-N prior to flash freezing in liquid nitrogen. Pcal_0546 crystals belong to the tetragonal space group I422, with the unit cell parameters a = b = 117.8, c = 89.4, α = β = γ = 90° (Supplementary Table S3). Data were collected at the beamline 19-ID and 19-BM of the Structural Biology Center, Advanced Photon Source, Argonne National Laboratory and processed using the program HKL3000 (26,27). The fluorescence spectrum recorded prior to data collection indicated the presence of Se and Fe in the crystallized protein. The structure was determined by the SADMR (singlet absorption detection of magnetic resonance) routine as implemented in the Auto-Rickshaw package (28). An initial model obtained from the search with the structure was rebuilt manually using the program COOT (29) and refined with the programs PHENIX (30) and REFMAC ver. 6.3.0 (31). Since the initial model indicated changes at the iron-sulfur cluster region in comparison to the SSO0001 structure, additional data were collected from the same crystal at the iron X-ray K absorption edge for anomalous Fourier difference maps validation (Supplementary Table S3). In addition, the analysis of the *2Fo-Fc, Fo-Fc* maps has revealed a strong peak in the active site (5.5 σ) suggesting the presence of a metal ion, which might be either Mg^2+^ (required for nuclease activity of Pcal_0546) or Na^+^ (present as 3.5 M Na-formate in the crystallization solution). Based on the analysis of the metal-binding site composition and geometrical arrangement of ligands, this density peak appears to represent a bound Mg^2+^ ion. The final model was refined to *R*_work_/*R*_free_ = 0.23/0.26, and it shows good geometry with no outliers in the Ramachandran plot. Data collection and refinement statistics are summarized in Supplementary Table S3.

## RESULTS

### The Cas4 protein Pcal_0546 contains a [2Fe-2S] cluster

**Figure 1.**
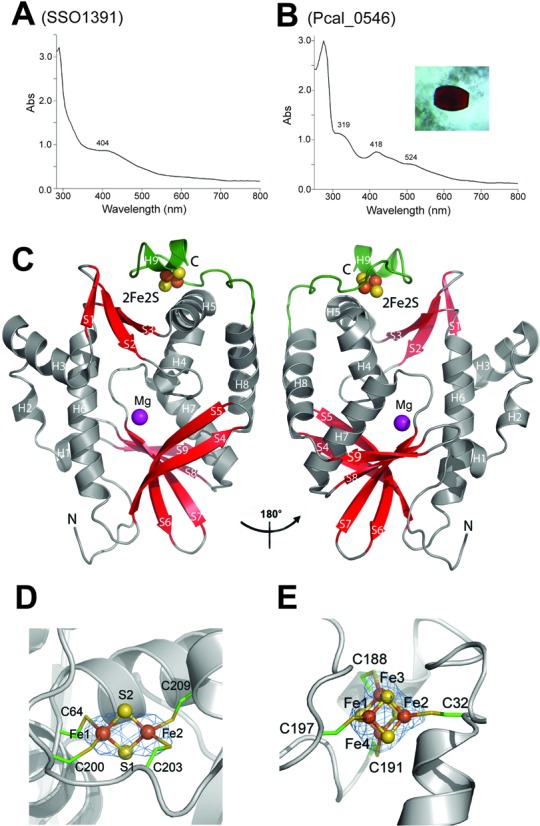
Cas4 protein Pcal_0546 contains a [2Fe-2S] cluster. (**A, B**) Absorption spectra of concentrated SSO1391 (A) and Pcal_0546 (B) proteins. The inset in (B) shows a photograph of the Pcal_0546 crystal. (**C**) Overall structure of the Pcal_0546 monomer: two views related by a 180° rotation. The protein core domain is colored in gray (helices) and red (strands), whereas the C-terminal domain is colored green and the position of the active site is indicated by the Mg^2+^ ion (magenta sphere). (**D**) Close-up view of the [2Fe-2S] cluster in Pcal_0546: iron anomalous difference Fourier map (blue mesh, contoured at 5 σ) indicates the position Fe. Iron atoms are shown as orange spheres, sulfur atoms as yellow spheres and the coordinating Cys residues are shown as sticks and labeled. (**E**) Orientation of the [4Fe-4S] cluster in SSO0001: iron anomalous difference Fourier map (blue mesh) of the SSO0001 [4Fe-4S] cluster (contoured at 6 σ). Color scheme is the same as for the panel D.

To reveal the biochemical diversity of Cas4 proteins, we cloned and purified four Cas4 proteins from different organisms including SSO1391 from *S. solfataricus*, Pcal_0546 from *P. calidifontis*, PAE1763 from *P. aerophilum* and Pisl_1722 from *P. islandicum*. Based on sequence, these proteins belong to either of two groups of Cas4 proteins (DUF83 and DUF911) and share low-to-medium sequence similarity to each other and to the recently characterized Cas4 nuclease SSO0001 (26–64% sequence identity) (Supplementary Figure S1). Although SSO0001 and the *Pyrobaculum* Cas4 proteins belong to the DUF83 family, based on phylogenetic analysis they form two distinct protein groups (Supplementary Figure S2). Like SSO0001 (19), purified SSO1391 had brown color in solution and showed the presence of one broad shoulder at 404 nm in its absorption spectrum suggesting the presence of an [4Fe-4S] cluster (Figure 1A). In contrast, the purified preparations of the Cas4 proteins Pcal_0546, PAE1763 and Pisl_1722 consistently showed a reddish or pink color in solution. Moreover, their absorption spectra were similar to that of plant-type ferredoxins or Rieske proteins containing a [2Fe-2S] cluster with a shoulder at 315–320 nm and two peaks at 416–420 nm and 517–524 nm (Figure 1B, Supplementary Figures S3A, S3B). The purified Cas4 proteins were submitted to crystallization trials, and Pcal_0546 produced red-colored crystals (Figure 1B). The structure of the selenomethionine substituted Pcal_0546 was solved to 2.65 Å resolution (Supplementary Table S3) and indeed revealed the presence of an [2Fe-2S] cluster associated with the C-terminal domain of the protein (Figure 1C).

The small C-terminal domain is formed by two α-helices (α4 and α8) and the long loop (Ser191-Pro207) coordinating the [2Fe-2S] cluster (Figure 1C). The identity of the cluster was confirmed by calculating anomalous Fourier difference maps calculated from data collected at the iron X-ray K absorption edge. The [2Fe-2S] cluster of Pcal_0546 is coordinated by the four conserved Cys residues (2.3 Å), one of which is located in the N-terminal part of the protein (Cys64), whereas the other three residues are at the C-terminus (Cys200, Cys203 and Cys209) (Figure 1C, Supplementary Figure S1). In the Pcal_0546 sequence, these cysteines are arranged in the same way as in SSO0001 (Supplementary Figure S1), where they coordinate a [4Fe-4S] cluster. Phylogenetic analysis of the DUF83 Cas4 proteins revealed that SSO0001 and Pcal_0546 belong to two different groups. The Pcal_0546 group also includes PAE1763 and Pisl_1722, which based on their absorption spectra appear to contain a [2Fe-2S] cluster too (Supplementary Figures S2, S3A, S3B). In the Pcal_0546 structure, the side chains of cluster-coordinating Cys residues have almost a rectangular planar arrangement (4.1 Å, 5.5 Å, 3.3 Å and 6.0 Å), whereas in the SSO0001 structure they form a pyramid with the N-terminal Cys32 on the top and the three C-terminal Cys (Cys188, Cys191 and Cys197) at the bottom (Figure 1D and E). The interatomic Fe-S and Fe-S_γ_ (Cys) distances of the Pcal_0546 [2Fe-2S] cluster (2.2 Å and 2.3 Å, respectively) are identical to that of the structurally characterized 2Fe-2S ferredoxins from *Anabaena* (32,33), but the Fe–Fe distance for Pcal_0546 is a bit longer: 3.1 Å compared to 2.7 Å in ferredoxins (Figure 1C).

The bacterial O_2_-sensing transcription factor FNR contains one [4Fe-4S] cluster under anaerobic conditions, which in the presence of O_2_ is quickly converted to a [2Fe-2S] cluster and then into an apo-form (without iron) resulting in a loss of site-specific DNA binding (34,35). In the *E. coli* FNR, the [2Fe-2S] cluster can be converted back to a [4Fe-4S] cluster by anaerobic incubation with Fe^2+^ and DTT (dithiothreitol) suggesting that these interconversions may represent a natural mechanism for the repair or biogenesis of [4Fe-4S] clusters (36). In contrast to the *E. coli* FNR, the [2Fe-2S] cluster of the purified Pcal_0546 was not affected by prolonged incubations in air, and its incubation under anaerobic conditions with Fe^2+^ and DTT or purification from anaerobically grown cells did not produce detectable amounts of [4Fe-4S] clusters (based on the absorption spectra of purified proteins; data not shown). The annotation of the *P. calidifontis* genome (e.g. in the PEDANT database) implies that this organism appears to be able to produce both [4Fe-4S] and [2Fe-2S] clusters, whereas *E. coli* is known to contain the biosynthetic pathways for both cluster types (37). These results suggest that the [2Fe-2S] cluster of Pcal_0546 is likely to be the natural form of the Fe-S cluster in this protein and is not a result of the oxidation of a [4Fe-4S] cluster. Thus, Cas4 proteins from different organisms can contain either a [4Fe-4S] or a [2Fe-2S] cluster.

### Nuclease activity of purified Pcal_0546 and SSO1391

**Figure 2.**
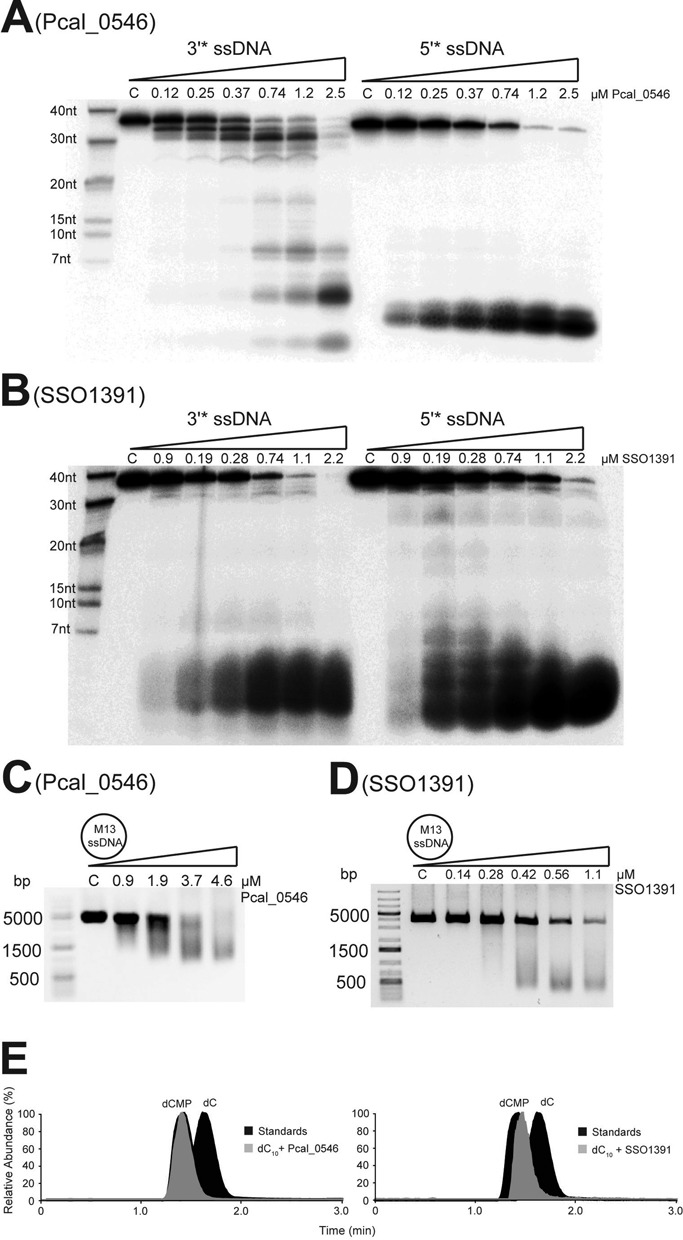
Cleavage of ssDNA by Pcal_0546 and SSO1391. (**A, B**) The 3′- or 5′-[^32^P]-labeled ssDNA was incubated without protein (lane C) or with indicated amounts of Pcal_0546 (A) or SSO1391 (B) for 15 minutes at 45ºC and analyzed by denaturing gel electrophoresis. (**C, D**) Endonuclease assays: the M13mp18 ssDNA (5 nM) was incubated at 45ºC without protein (lane C) or in the presence of indicated amounts of Pcal_0546 (C) or SSO1391 (D) and the reaction products were analyzed by agarose gel electrophoresis and SYBR Green staining. (**E**) Mass spectrometry analysis of the ssDNA cleavage products generated by Pcal_0546 and SSO1391. Relative abundance of ions and retention times corresponding to dC (m/z 226.0834) and dCMP (m/z 306.0497) are shown for standards (black) and products of Pcal_0546 or SSO1391 mediated cleavage of a 10 nt long oligonucleotide (gray).

Purified Pcal_0546 and SSO1391 demonstrated metal-dependent nuclease activity against [^32^P]-labeled ssDNA substrates (Figure 2). Both proteins were active in a pH range of 7–9 with maximal activity at pH 8.0 in the presence of Mn^2+^ followed by Mg^2+^, Co^2+^ and Ni^2+^, whereas Ca^2+^, Cu^2+^ and Zn^2+^ did not support nuclease activity (Supplementary Figures S3C–S3F). The temperature profile of the nuclease activity of Pcal_0546 was similar to that of SSO0001 (19) with maximal activity in the range of 45–70ºC (Supplementary Figure S3G). With the 5′-labeled ssDNA substrate, Pcal_0546 produced a single product (3 nt), whereas a series of products with varying chain lengths was generated from the 3′-labeled ssDNA substrate indicating that this protein cleaves ssDNA in the 5′ → 3′ direction (Figure 2A). In contrast, SSO1391 cleaved ssDNA both in the 5′ → 3′ and 3′ → 5′ directions based on the formation of multiple products both with the 3′- and 5′-[^32^P]-labeled substrates (Figure 2B). Both Pcal_0546 and SSO1391 also exhibited Mn^2+^-dependent endonuclease activity against the circular ssDNA of M13mp18 as substrate (Figure 2C, D). LC-MS analysis revealed that the ssDNA cleavage products of both Pcal_0546 and SSO1391 contain 5′-hydroxyls and 3′-phosphates (Figure 2E). This is consistent with the types of DNA cleavage products formed by SSO0001, whereas the characterized RecB-like nucleases are known to produce the 5′-phosphate and 3′-hydroxyl containing products (21,38).

**Figure 3.**
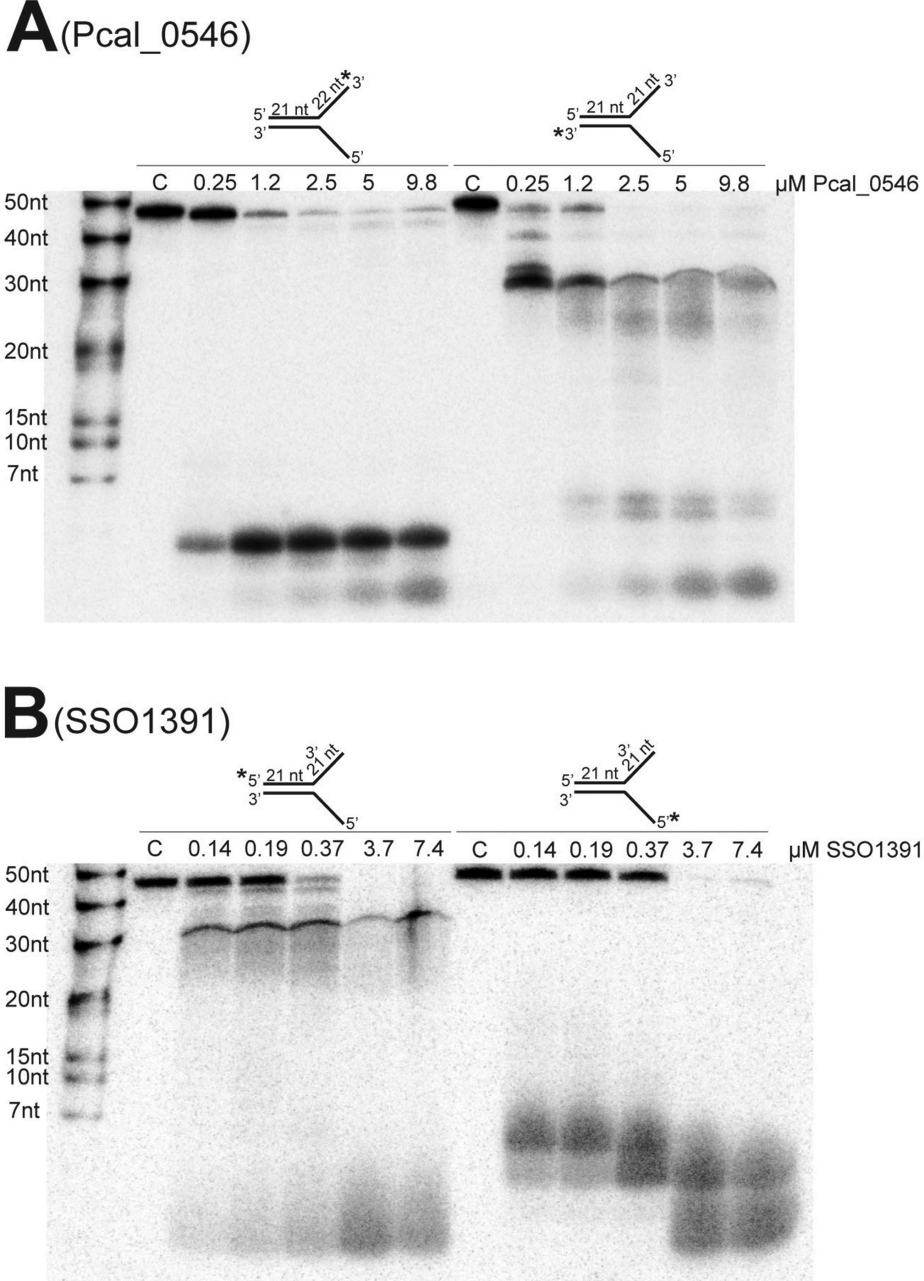
Unwinding and cleavage of splayed arm substrates by Pcal_0546 and SSO1391. (**A, B**) Cleavage of splayed arm substrates by the indicated amounts of Pcal_0546 (A) or SSO1391 (B) incubated for 45 min (A) or 15 min (B) and analyzed by denaturing gel electrophoresis. In all substrates, one strand is [^32^P]-labeled at the 3′-end (A) or 5′-end (B) (indicated by asterisks), and the length of ssDNA overhangs is indicated on the substrate models (nt). Lane C shows incubation without protein.

Splayed arm-like (SA) DNA structures are useful model substrates for the RecB-like nuclease complexes, which catalyze ATP-dependent dsDNA unwinding and ATP-independent ssDNA cleavage reactions (20,22,39,40). Both Pcal_0546 and SSO1391 cleaved these substrates (42 or 59–60 nt long) with the formation of short labeled products suggesting that they were able to unwind the double-stranded part of DNA substrate and continue the cleavage of the labeled DNA strand (Figure 3, Supplementary Figure S3M, S3N). These assays also revealed the transient accumulation of reaction intermediates consistent with the enzyme potentially pausing when it reaches the dsDNA area (20–30 nt in Figure 3; 30–40 nt in Supplementary Figure S3M, S3N). Compared to the cleavage of ssDNA substrates, the unwinding and cleavage of SA substrates by both proteins were slower and required higher (5–7 times) protein amounts suggesting that the SA unwinding step is limiting in this reaction. Similar to SSO0001, unwinding and cleavage of SA substrates were not stimulated by the addition of ATP (up to 2 mM) suggesting that their DNA unwinding activity is ATP-independent (Supplementary Figure S3O, S3P). It is possible that, *in vivo*, the activity of Cas4 proteins can be regulated by binding to other Cas or non-Cas proteins.

### Overall structure of Pcal_0546 and its active site

**Figure 4.**
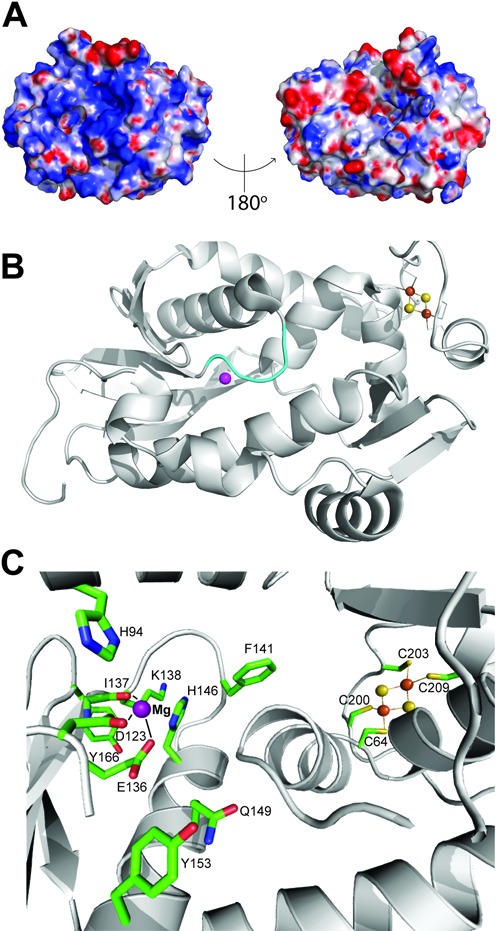
Structure of the active site of Pcal_0546. (**A**) Surface charge distribution of Pcal_0546 with a blue (positively charged) to red (negatively charged) gradient. (**B**) The orientation of the loop (138–142) located at the bottom of the active site of Pcal_0546 (as opposed to the open active site in SSO0001). The Mg^2+^ ion is represented by the magenta sphere. (**C**) Close-up view of the Pcal_0546 active site. Residues are shown as sticks and labeled, the Mg^2+^ ion is shown as a purple sphere, and the [2Fe-2S] cluster as orange and yellow spheres.

The central core structure of Pcal_0546 adopts an αβ fold, which is similar to that of SSO0001, with a central six stranded V-shaped β-sheet surrounded by eight α-helices (Figure 1C). The fold analysis using the Dali server confirms that SSO0001 is the closest homolog of Pcal_0546 with Z-score of 11.7 (r.m.s.d. of 3.5 Å; 26% sequence identity). Similar to SSO0001, the Pcal_0546 structure shows the presence of two distinct domains: the core domain and the Fe-S cluster-binding C-terminal domain. The core domain contains the active site, which is located in a well defined cavity formed by the α-helices H6, H7 and the β-strand S3. The active site of SSO0001 has two openings, which have been proposed to represent the substrate entrance and product release channels (19). Likewise, the structure of Pcal_0546 revealed the presence of two pockets on opposite sides of the protein with different surface charge distribution representing the bottom opening (the left panel of Figure 4A) and the active site cleft (the right panel of Figure 4A; Supplementary Figure S4A). In contrast to SSO0001, the bottom opening of the Pcal_0546 active site is almost closed by the flexible loop Ile138-Lys142 and likely represents the closed state of the potential product release channel (Figure 4B).

The metal ion bound in the active site was assigned as an Mg^2+^ ion based on the distances (2.1–2.4 Å) to coordinating residues. The Mg^2+^ ion is located 22.4 Å away from the [2Fe-2S] cluster suggesting that the cluster is not likely to be directly involved in catalysis (Figure 4C). The metal ion is coordinated by the Asp123 (2.4 Å) and Glu136 (2.4 Å) residues of the RecB-nuclease motifs II and III, the main chain carbonyl oxygen of Ile137 (2.7 Å) and a water molecule (2.4 Å) (Figure 4C). Since Pcal_0546 uses divalent metal cations for activity (Mn^2+^ or Mg^2+^), we propose that the side chain of the conserved His94 might provide the fourth ligand for these ions (4.2 Å from Mg^2+^ in this Pcal_0546 structure) (Figure 4C). Similar divalent metal ion coordination was observed in SSO0001 (His62, Asp99, Glu113 and Ile114) and *E. coli* RecB (19,21).

Size-exclusion chromatography indicated that Pcal_0546 exists mostly as a monomer in solution (28.3 kDa; predicted molecular mass 27.1 kDa), whereas SSO1391 forms a dimer (67 kDa; predicted molecular mass 35.9 kDa) (Supplementary Figure S3K). In contrast, the crystal structure of the Cas4 protein SSO0001 revealed a decameric toroid formed by five tightly bound dimers (19). Superimposition of structures of Pcal_0546 and SSO0001 reveals that, despite having similar overall architectures and active sites with a good overlap of the metal ions, the two structures differ considerably as shown by a large r.m.s.d. of 3.5 Å (over 164 residues) (Supplementary Figure S4B, S4C). One notable difference is the presence of an N-terminal extension (26 amino acids) in Pcal_0546, which covers the long α-helix H5 that is involved in dimerization of SSO0001 (Supplementary Figure S4B). This flexible N-terminal extension can potentially prevent the formation of Pcal_0546 dimers and higher oligomers (Supplementary Figure S4D). Another difference is associated with the presence of a three stranded anti-parallel β-sheet in Pcal_0546 (Supplementary Figures S1, S2 and S3), which can potentially contribute to the stabilization of the [2Fe-2S] cluster domain of this protein (Figure 1C, Supplementary Figure S4). In addition, in the structure of SSO0001 the active site bottom loop (116–126) is shifted away thereby opening the product exit channel, whereas Pcal_0546 has a shorter loop (138–142) oriented toward the active site (Figure 4B). Finally, the C-terminal domains of these proteins accommodate different Fe-S clusters, so that only one of the four cluster-coordinating Cys residues in the two proteins is aligned closely (Cys64 in Pcal_0546 and Cys32 in SSO0001).

### Site-directed mutagenesis of Pcal_0546 and SSO1391

**Figure 5.**
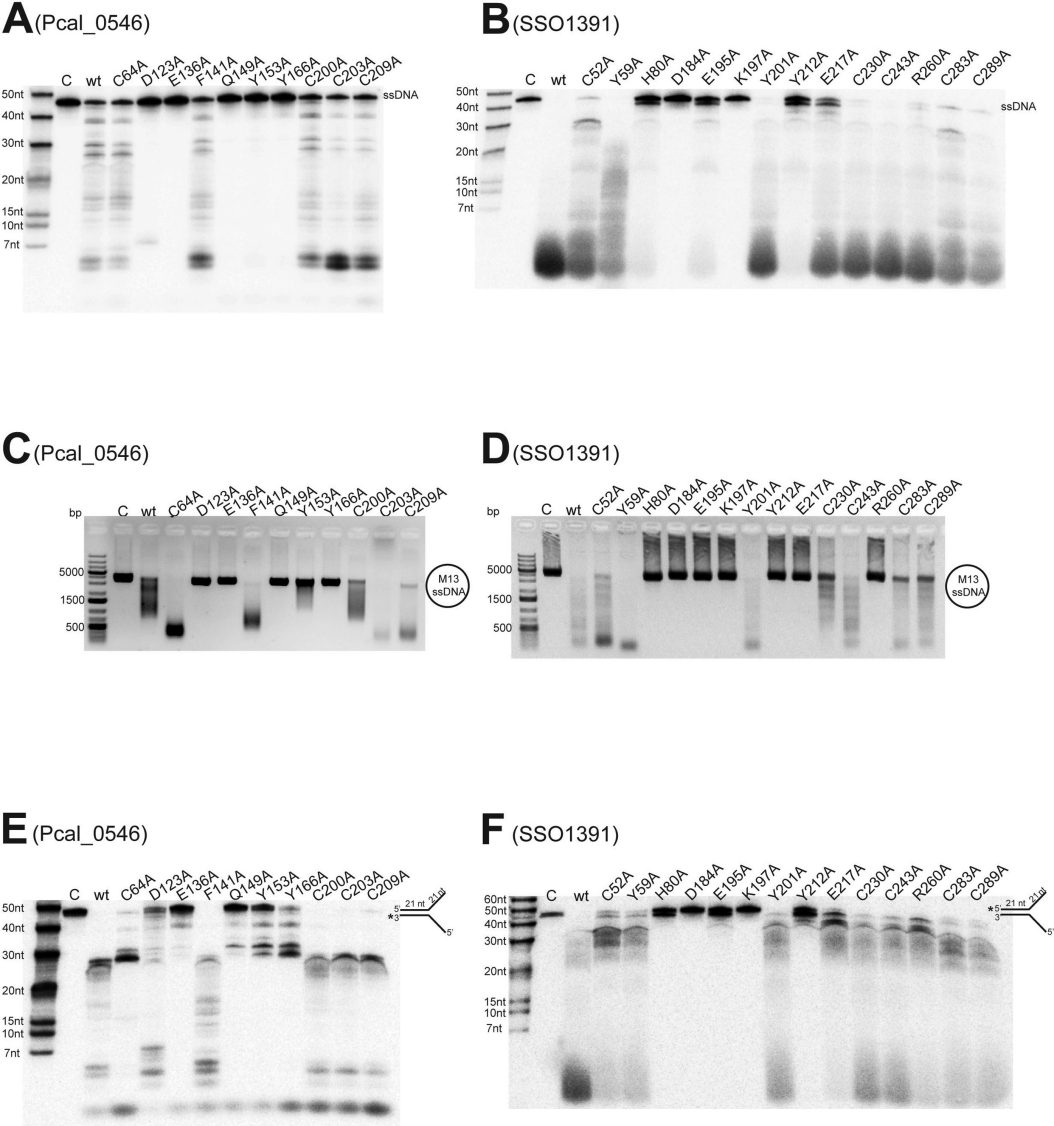
Site-directed mutagenesis of Pcal_0546 and SSO1391. (**A, B**) Cleavage of ssDNA by the purified wild type (wt) or mutant Pcal_0546 (A) or SSO1391 (B). Reactions contained 246 nM Pcal_0546 or 742 nM SSO1391 (15 min incubation, denaturing gel electrophoresis). (**C, D**) Cleavage of the circular ssDNA of M13mp18 by Pcal_0546 (C) or SSO1391 (D). Reactions contained 2.2 μM Pcal_0546 wt or mutant proteins (1.1 μM for Cys mutants) or 1.4 μM SSO1391 and were incubated for 10 min at 45°C (C) or for 20 min at 40°C (D) (agarose gel electrophoresis). (**E, F**) Cleavage of splayed arm substrates by Pcal_0546 (E) or SSO1391 (F). Reactions contained 1.9 μM Pcal_0546 or SSO1391 and were incubated for 45 min (E) or 30 min (F) at 45°C (denaturing gel electrophoresis). In all gels lane C shows incubation without protein.

The crystal structure of Pcal_0546 provides insights into the active site and molecular mechanisms of nuclease activity of Cas4 nucleases. Site-directed mutagenesis was used to identify the residues of Pcal_0546 and SSO1391 required for substrate unwinding and cleavage (Figure 5). Sequence alignments of Pcal_0546 and SSO1391 with other proteins from the DUF83 and DUF911 families revealed over 35 conserved residues including the RecB motif residues and the Fe-S cluster coordinating cysteines (Supplementary Figure S1). As expected, alanine replacement mutagenesis of the residues of the RecB motifs II, III and QhXXY, in both Pcal_0546 (Asp123, Glu136, Gln149 and Tyr153) and SSO1391 (Asp184, Glu195, Lys197 and Tyr212), produced almost inactive proteins in both exonuclease and endonuclease reactions (Figure 5). In the reaction of unwinding/cleavage of SA substrates, the SSO1391 RecB motif mutants were also inactive, whereas the RecB mutant proteins of Pcal_0546 showed detectable activity (due to the higher protein load required for this assay) (Figure 5E, F).

The RecB-like nucleases containing an iron-sulfur cluster have been termed ‘iron staple’ nucleases, because the intact Fe-S cluster is required for dsDNA end-binding and DNA break processing by the AddAB nuclease complex from *B. subtilis* (41). Similarly, disruption of the Fe-S cluster by mutagenesis inactivated the Cas4 nuclease SSO0001 (19,41). Alanine replacement mutagenesis of the conserved Cys residues coordinating the Fe-S cluster in both Pcal_0546 (Cys64, Cys200, Cys203 and Cys209) and SSO1391 (Cys52, Cys283 and Cys289) resulted in the production of colorless proteins without absorbance in the 320–550 nm range indicating that they have no assembled Fe-S clusters. Surprisingly, both Pcal_0546 and SSO1391 Cys-to-Ala mutant proteins showed wild type level nuclease activity in both exonuclease and endonuclease reactions, as well as in the unwinding/cleavage of SA substrates (Figure 5).

The presence of high catalytic activity in the Cys mutant proteins of Pcal_0546 and SSO1391 suggests that the nuclease core of these proteins retains the catalytically active conformation in the absence of intact Fe-S clusters. For Pcal_0546, this might be due to the presence of an additional β-sheet (Supplementary Figures S1, S2 and S3), which is absent in SSO0001 (Figure 1C). This 3-strand anti-parallel β-sheet is located between the core and C-terminal domains of Pcal_0546 and contains several conserved residues (Leu39, Ile43, Tyr 44, Lys77, Leu78, Leu79), which can interact with the conserved Phe205 and Ile208 of the C-terminal domain or with the residues of the core domain (2.6–4.2 Å). These multiple hydrophobic and ionic/polar interactions can potentially stabilize both protein domains making the core nuclease domain more resistant to the disruption of the Fe-S cluster.

Alanine replacement mutagenesis of most non-RecB motif or non-conserved residues of both Pcal_0546 (Phe141) and SSO1391 (Tyr59, Tyr201, Glu217, Cy230 and Cys243) had no negative effect on the nuclease activities of these proteins, whereas the Pcal_0546 Y166A and SSO1391 H80A were inactive (Figure 5). In Pcal_0546, the non-conserved Phe141 is located at the bottom of the active site partially blocking the potential product exit channel (Figure 4C). Therefore, removal of this bulky side chain can stimulate nuclease activity through the increase of the active site space and product removal. The Pcal_0546 Tyr166 is also conserved in SSO0001 (Tyr148), where it was also essential for activity and is proposed to play a role in DNA coordination within the active site (19). In addition, the structures of both Pcal_0546 and SSO0001 show the presence of a conserved Gln residue in the active site close to the bound metal ion (Gln149 and Gln131, respectively). However, while the SSO0001 Q131A mutant protein retained significant nuclease activity (19), the Pcal_0546 Q149A was inactive in both exonuclease and endonuclease reactions (Figure 5). Interestingly, the SSO1391 E217A and R260A proteins showed high exonuclease activity, but they were inactive in the endonuclease reaction suggesting that these residues are critical for the endonucleolytic substrate cleavage by the DUF 911 proteins (Figure 5B, D).

## DISCUSSION

Iron-sulfur clusters are known to be important cofactors of many nucleic acid enzymes and binding proteins that function in DNA repair, replication, recombination, transcription, regulation, genome protection and include different nucleases, helicases, glycosylases, RNA polymerases, RNA methyltransferases and transcription factors (42–44). In these proteins, iron-sulfur clusters can play different roles including structural, DNA binding, DNA translocation and unwinding, protein interaction (complex assembly) and redox sensing (42). It is striking that all known nucleic acid processing metalloenzymes contain [4Fe-4S] or [3Fe-4S] clusters with two exceptions: the rotavirus RNA-binding 2Fe-2S-protein NSP5 and the mono-iron rubredoxin-like Fe-Cys_4_ center in the rare-cutting restriction endonuclease NotI from *Nocardia otitidiscaviarum* (45,46). Thus, the Cas4 nuclease Pcal_0546 characterized in our work represents the first DNA processing enzyme with a [2Fe-2S] cluster. Based on the absorption spectra of purified proteins, the Cas4 proteins PAE1763 and Pisl_1722 are also likely to contain a [2Fe-2S] cluster (Supplementary Figure S3). Based on our results with Pcal_0546, its [2Fe-2S] cluster appears not to be an intermediate of the degradation of a [4Fe-4S] cluster. Presently, it is not clear why Pcal_0546 and probably other Cas4 proteins from *Pyrobaculum* contain a [2Fe-2S] cluster, so further biochemical studies with other Cas4 proteins are required. Although the optimal growth temperature of *Pyrobaculum* is higher than that of *Sulfolobus* (100°C and 75–80°C, respectively), there is no experimental evidence to suggest that 2Fe-2S clusters have higher thermostability compared to 4Fe-4S clusters (47).

The structure of Pcal_0546 revealed the presence of a second β-sheet located between the RecB nuclease active site and the [2Fe-2S] cluster (Figure 1C). This β-sheet is absent in SSO0001, and its C-terminal [4Fe-4S] cluster domain is directly connected to the nuclease domain (19). Here, it forms part of the active site wall making the active site sensitive to perturbations in the iron-sulfur cluster domain, and therefore the disruption of the Fe-S cluster staple abolished nuclease activity of SSO0001 (19). Since the Pcal_0546 Cys mutant proteins were catalytically active (Figure 5), we propose that this β-sheet provides additional interactions stabilizing both nuclease and [2Fe-2S] domains of this protein. Therefore, the disruption of the cluster by alanine replacement of the coordinating Cys residues had no negative effect on the nuclease activity of Pcal_0546 (Figure 5). Moreover, reduced activity of the SSO1391 Cys-to-Ala mutant proteins (C52A, C283A and C289A) in the SA unwinding/cleavage reaction suggests that its Fe-S cluster might also contribute to DNA unwinding (Figure 5F).

Another remarkable difference between the characterized Cas4 proteins is associated with their oligomeric state. The recently characterized Cas4 protein SSO0001 was found to form a toroid decamer from five tightly bound dimers (19), whereas Pcal_0546 exists as a monomer and SSO1391 as a dimer in solution (this work). Pcal_0546 shares high sequence similarity (55% sequence identity) to the Cas4 protein TTX_1548 from *Thermoproteus tenax*, which has been shown to form a tight complex with the *T. tenax* Cas1, Cas2, and Csa1 proteins *in vitro* (48). Therefore, we propose that Pcal_0546 might also form a complex with the corresponding Cas1, Cas2 and Csa1 proteins from *P. calidifontis in vivo* that is in line with the monomeric state of the purified Pcal_0546. As part of this complex, Pcal_0546 can generate ssDNA overhangs as potential intermediates for new spacer generation by Cas1 and Cas2 proteins. Since many CRISPR containing genomes (including *P. calidifontis* and *S. solfataricus*) encode multiple Cas4 genes, these proteins might also contribute to other CRISPR processes and non-CRISPR pathways (e.g. DNA repair). The possibility for an alternative (non-CRISPR) role of Pcal_0546 in nucleic acid metabolism is consistent with the location of its gene and homologous genes in other *Pyrobaculum* species (Pars_0755 and PAE1763) outside of the Cas operons. Thus, our work has revealed significant structural and biochemical diversity of Cas4 nucleases, which can have different oligomeric states (monomers, dimers and decamers), contain different iron-sulfur clusters ([2Fe-2S] or [4Fe-4S]) and cleave ssDNA in different directions.

## ACCESSION NUMBERS

Coordinates and structure factors for the Pcal_0546 structure have been deposited in the Protein Data Bank with the accession code 4R5Q.

## SUPPLEMENTARY DATA

Supplementary Data are available at NAR Online.

SUPPLEMENTARY DATA
