# Supplementary material for: The CRISPR-associated Cas4 protein Pcal_0546 from Pyrobaculum calidifontis contains a [2Fe-2S] cluster: crystal structure and nuclease activity
Source: Nucleic Acids Res. 2014 Sep 8;42(17):11144–55. doi: 10.1093/nar/gku797 (PMC4176176; doi:10.1093/nar/gku797)
Supplement: SUPPLEMENTARY DATA [file supp_42_17_11144__index.html]

The CRISPR-associated Cas4 protein Pcal\_0546 from Pyrobaculum calidifontis contains a [2Fe-2S] cluster: crystal structure and nuclease activity — The CRISPR-associated Cas4 protein Pcal\_0546 from Pyrobaculum calidifontis contains a [2Fe-2S] cluster: crystal structure and nuclease activity — SUPPLEMENTARY DATA 

# The CRISPR-associated Cas4 protein Pcal\_0546 from *Pyrobaculum calidifontis* contains a [2Fe-2S] cluster: crystal structure and nuclease activity

## SUPPLEMENTARY DATA

**Files in this Data Supplement:**

- SUPPLEMENTARY DATA
