# Supplementary material for: The CRISPR-associated Cas4 protein Pcal_0546 from Pyrobaculum calidifontis contains a [2Fe-2S] cluster: crystal structure and nuclease activity
Source: Nucleic Acids Res. 2014 Sep 8;42(17):11144–55. doi: 10.1093/nar/gku797 (PMC4176176; doi:10.1093/nar/gku797)
Supplement: SUPPLEMENTARY DATA [file supp_gku797_nar-01486-h-2014-File007.pdf]

SUPPLEMENTARY DATA

Supplementary Figure Legends

Supplementary Figure S1. Sequence alignment of DUF83 (**A**) and DUF911 (**B**) Cas4 family proteins. Conserved residues are highlighted in blue and the RecB-like motif residues (I: E; II: D; III: EhK, and QhXXY) are boxed in red. The secondary structure elements of Pcal_0546 are shown above the alignment, whereas the secondary structure elements of SSO0001 are shown below (A). Pcal_0546 (A) and SSO1391 (B) residues mutated to Ala are numbered and marked with triangles. The proteins compared are: (**A**) Pcal_0546 (UniProt ID A3MTK6) from *P. calidifontis*, Pisl_1722 (A1RV91) from *P. islandicum*, PAE1763 (Q8ZWJ5) from *P. aerophilum*, and SSO0001 (Q97TX9) from *S. solfataricus*; (**B**) SSO1391 (Q97YD4) from *S. solfataricus*, Pcal_1274 (A3MVN1) from *P. calidifontis*, and HM1_0519 (B0TFX6) from *Heliobacterium modesticaldum*.

Supplementary Figure S2. Phylogenetic analysis of the DUF83 Cas4 proteins. Phylogenetic tree representing the relationships among members of the DUF83 family of proteins: MetMK1DRAFT_00006860 (UniProt ID H2C1R3) from *Metallosphaera yellowstonensis*, Desmu_0121 (E8R707) from *Desulfurococcus mucosus*, Igag_1219 (E0SP98) from *Ignisphaera aggregans*, Saci_0274 (Q4JBY9) from *S. acidocaldarius*, SSO0001 (Q97TX9) from *S. solfataricus*, PAE1763 (Q8ZWJ5) from *P. aerophilum*, Pars_0755 (A4WIX4) from *P. arsenaticum*, Pogu_1588 (H6Q986) from *P. oguniense*, Tneu_0740 (B1YD16) from *P. neutrophilum*, Pisl_1722 (A1RV91) from *P. islandicum*, Pcal_0546 (A3MTK6) from *P. calidifontis*, TUZN_1991 (F2L4U5) from *Thermoproteus*

uzoniensis, and TTX_1548 (G4RKS8) from *Thermoproteus tenax*. Bootstrap values are indicated at the nodes.

Supplementary Figure S3. Biochemical characterization of Cas4 nucleases. (**A, B**) Absorption spectra of concentrated PAE1763 (A) and Pisl_1722 (B) proteins. (**C, D**) Effect of divalent metal ions on nuclease activity of Pcal_0546 (C) or SSO1391 (D). Either 3′-[^{32}P]-labeled ssDNA (C) or 5′-[^{32}P]-labeled ssDNA (D) was incubated with 0.5 μM of Pcal_0546 in 50 mM Tris-HCl, pH 8, 10 mM KCl and 1 mM metal for 20 minutes at 45°C or 4.6 μM SSO1391 in 50 mM Tris-HCl, pH 8, 5 mM KCl and 2 mM metal for 1 hour at 50°C. Lane C shows incubation without protein. (**E, F**) Effect of pH on ssDNA cleavage by Pcal_0546 (E) or SSO1391 (F). 3'- labelled (E) or 5'- labelled (F) ssDNA substrates were incubated with 0.5 μM (E) or 4.6 μM (F) protein for 20 min(E) or 1 hour(F) at 45°C (E) or 50°C (F) in 50 mM buffer and the presence of 10 mM (E) or 50 mM (F) KCl, 2 mM (E), or 5 mM (F) $MgCl_2$, 1 mM DTT (E) and reaction products were analysed by denaturing PAGE. (**G**) Effect of temperature on ssDNA cleavage by Pcal_0546. 3'- labelled ssDNA substrates were incubated with 1.2 μM protein for 20 min at the indicated temperature in 50 mM Tris-HCl, pH 8 in the presence of 10 mM KCl, 5 mM $MgCl_2$, and 1 mM DTT. (**H, I, J**) Effect of $MgCl_2$ (H), KCl (I), and $CoCl_2$ (J) concentration on ssDNA cleavage by Pcal_0546 (H, I) or SSO1391 (J). Substrates were incubated with 2.5 μM Pcal_0546 for 30 min at 45°C in 50 mM Tris-HCl (pH 8) (H, I) or MES (pH 6.5) (J) in the presence of 10 mM (H) or 5 mM (J) KCl (or as indicated), 10 mM $MgCl_2$ (or as indicated), or the indicated amount of $CoCl_2$ (J) and 1 mM DTT (H, I). **(K)** Effect of $MgCl_2$ concentration on endonucleolytic ssDNA cleavage by Pcal_0546.

Substrate was incubated for 10 min at 45°C with 3.7 µM Pcal_0546 in 50 mM Tris-HCl (pH 8), indicated amount of $MgCl_2$ and 5 nM ssDNA of the M13mp18 phage. Reaction products were analyzed by agarose gel electrophoresis and SYBR Green staining. (**L**) Size exclusion elution profiles of Pcal_0546 and SSO1391 using Superdex S200 10/300 GL. The elution volume is indicated. (**M, N**) Unwinding and cleavage of splayed arm substrates by Pcal_0546 and SSO1391. Cleavage of splayed arm substrates by the indicated amounts of Pcal_0546 (M) or SSO1391 (N) after 45 minutes. In all substrates, one strand is [^{32}P]-labelled at the 3'-end (indicated by asterisks), and the length of ssDNA overhangs is indicated on the substrate models (nt). Lane C shows incubation without protein. (**O, P**) Effect of ATP addition (0-2 mM) on cleavage of splayed arm substrates by 1.2 µM Pcal_0546 (O) or 371 nM SSO1391 (P) at 45°C. Lane C: incubation without protein.

Supplementary Figure S4. Structural characterization of Pcal_0546. (**A**) View of Pcal_0546 surface (positively charged (blue) to negatively charged (red) gradient) showing the cavity containing the active site (Mg^{2+} ion shown as a green sphere). (**B**) Structural superimposition of Pcal_0546 (green) and SSO0001 (orange, 4IC1) showing the position of the Pcal_0546 N-terminal extension (with the short α-helices H1 and H2) over the long α-helix H6 (α-helix H5 in SSO0001). The protein ribbons are colored green (Pcal_0546) and orange (SSO0001). The metal ions (magenta/pink spheres) and Fe-S clusters (orange/yellow spheres) are also shown. (**C**) Structural superimposition of the catalytic residues and metal ions in the active sites of SSO0001 (4IC1), and Pcal_0546. The protein ribbons and side chain carbons are colored green (SSO0001) and orange

(Pcal_0546). The labelled residues are K115 in SSO0001 and K138 in Pcal_0546. The SSO0001 metal ion (magenta colored sphere) is labeled as Mn^{2+}, whereas the metal ion of Pcal_0546 (pink colored sphere) is labeled as Mg^{2+}. (**D**), The SSO0001 dimer (4IC1) showing tight packing with multiple interactions between two protomers (I and II), which involve the α-helices H5. Dimerization of Pcal_0546 is prevented by the presence of the N-terminal extension over the corresponding α-helix H6 (shown on panel B).

Supplementary Figure S5. Nuclease activity of the wild type and mutant Pcal_0546 and SSO1391. Quantification of linear ssDNA cleavage (**A, B**), circular ssDNA cleavage (**C, D**), and splayed arm unwinding (**E, F**) of Pcal_0546 (**A, C, E**) or SSO1391 (**B, D, F**) wt and mutant proteins as presented in Figure 5. The values were obtained using ImageQuantTL (7.0) and Image Lab (4.1) software. Percentage of unwinding was determined by quantifying the percentage of products shorter than 21 nt in length.

Supplementary Figure S1

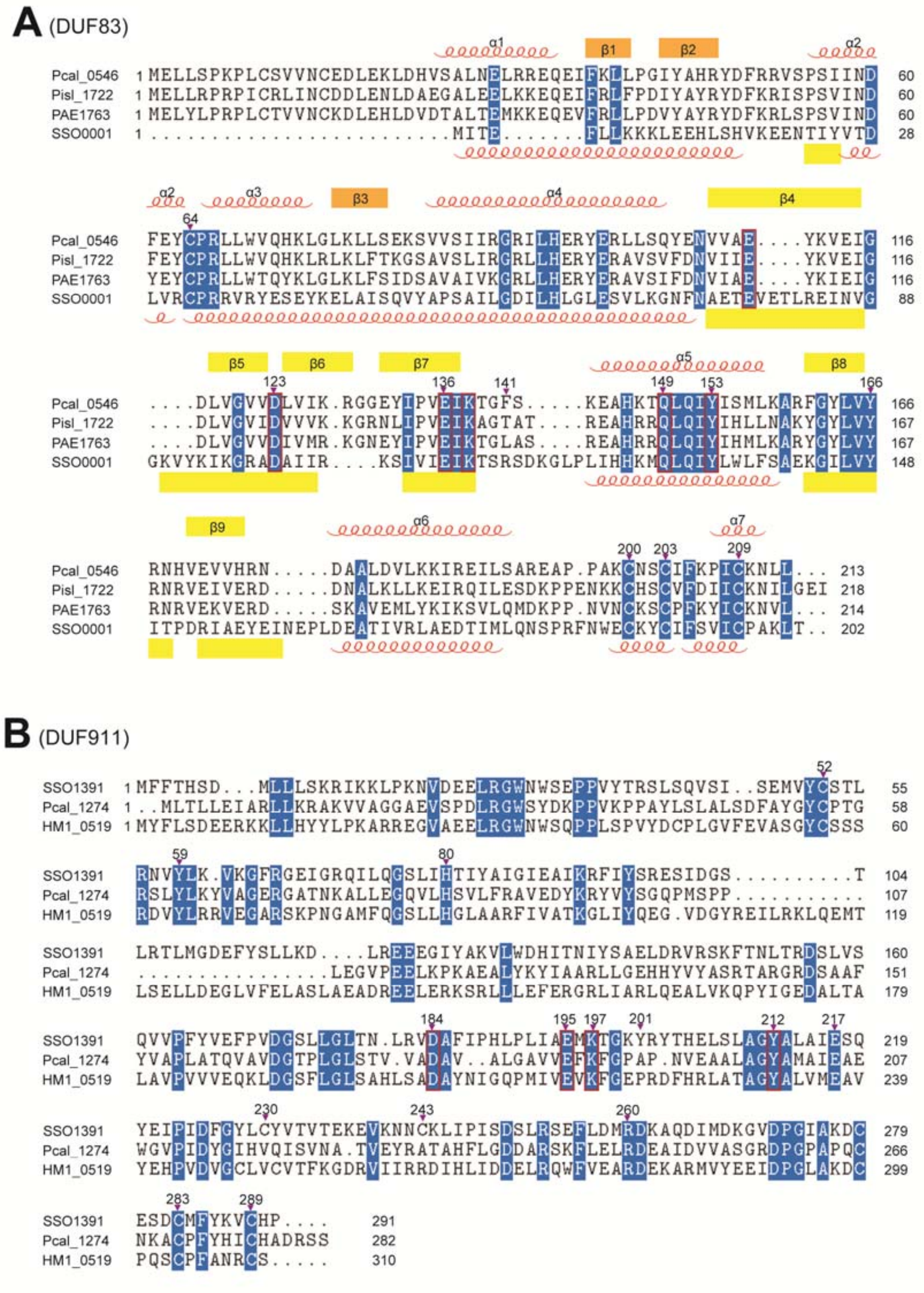

Supplementary Figure S2

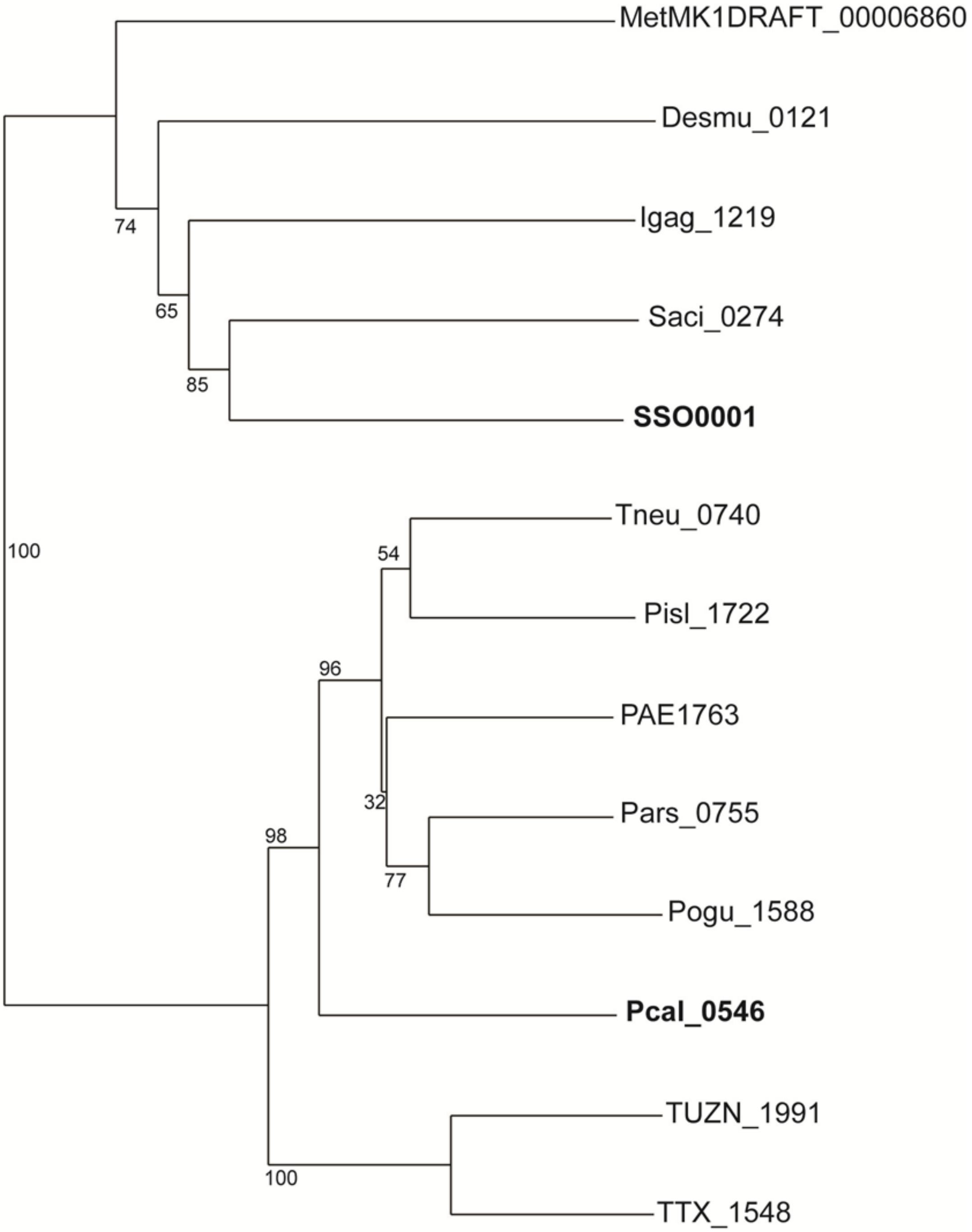

Supplementary Figure S3

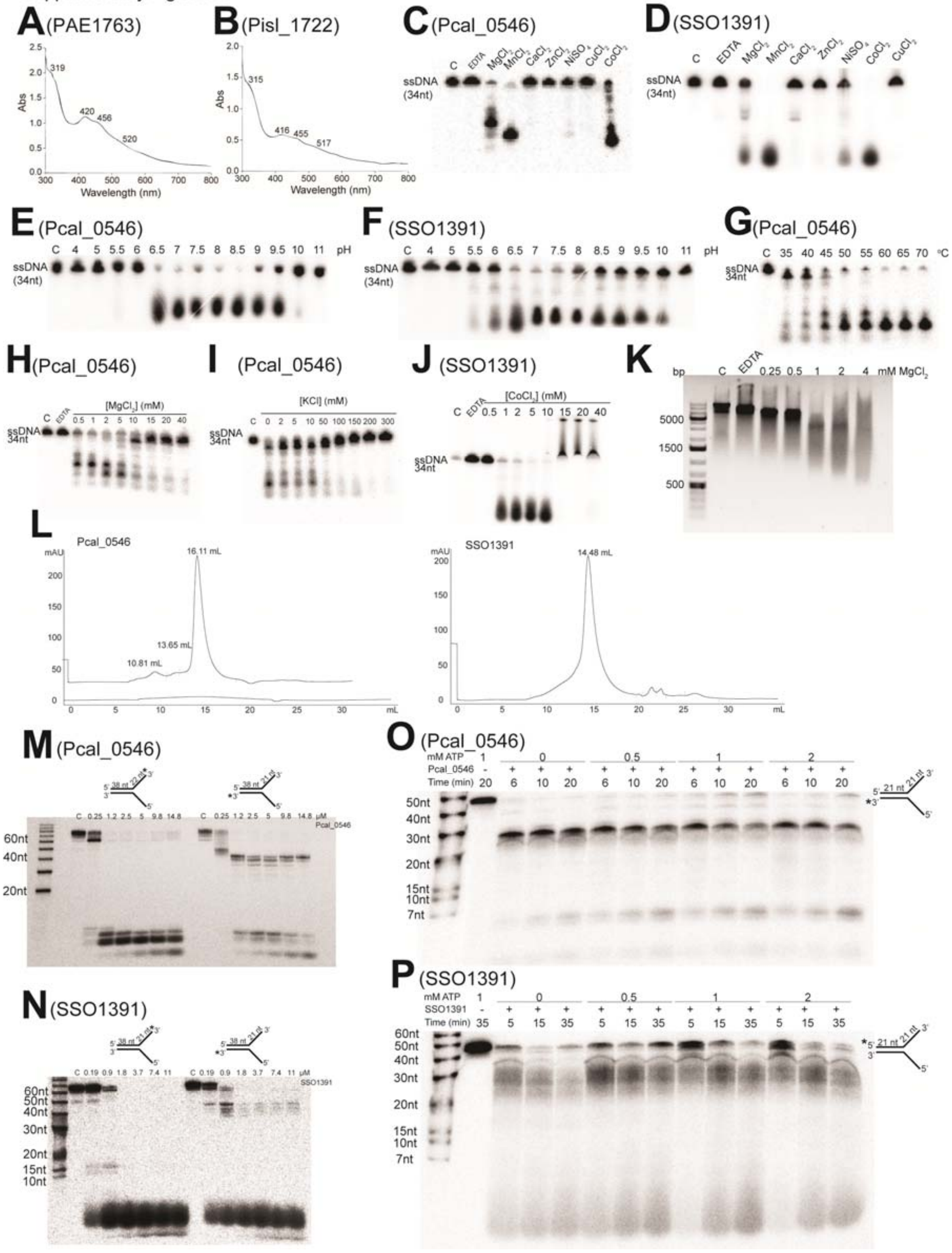

Supplementary Figure S4

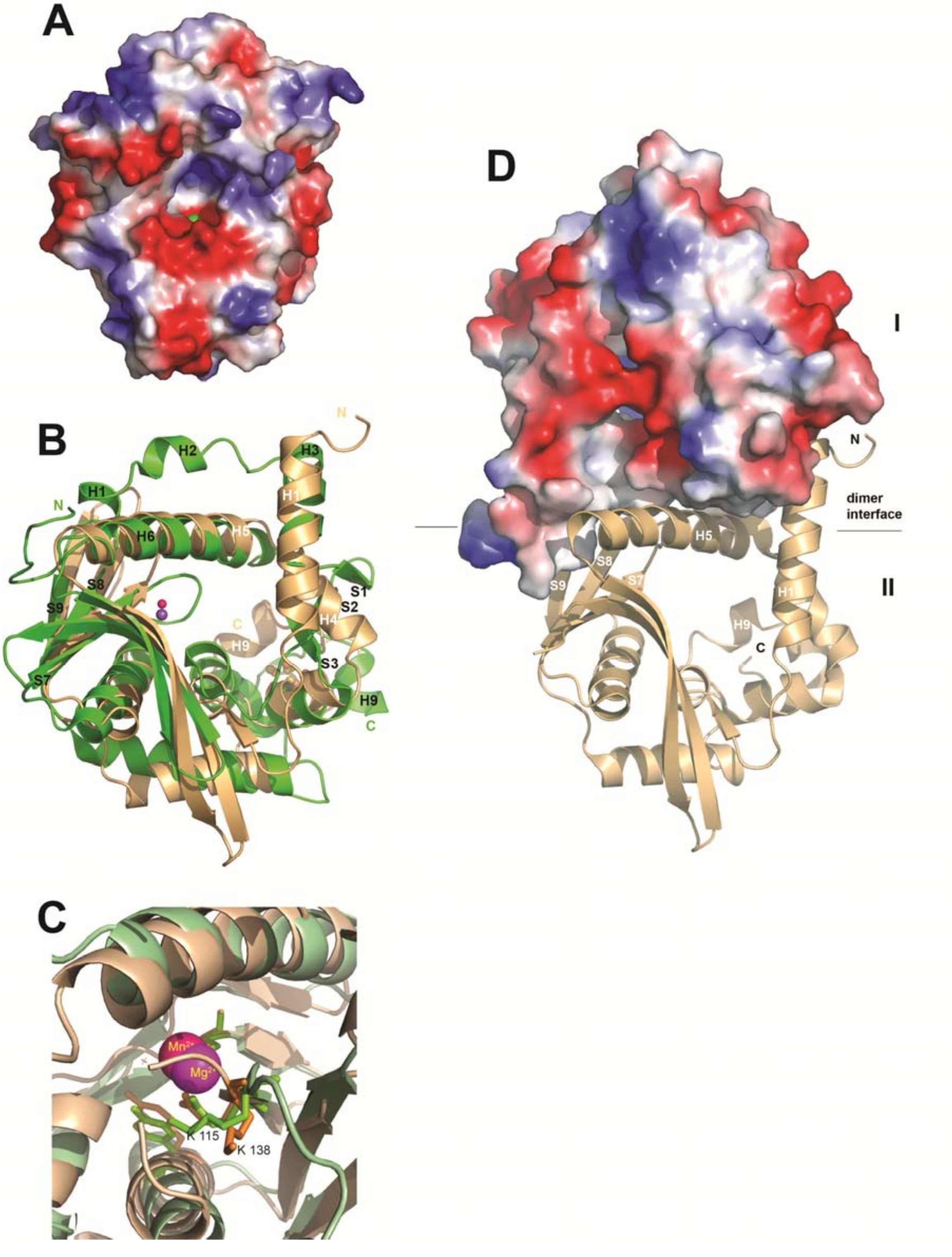

Supplementary Figure S5

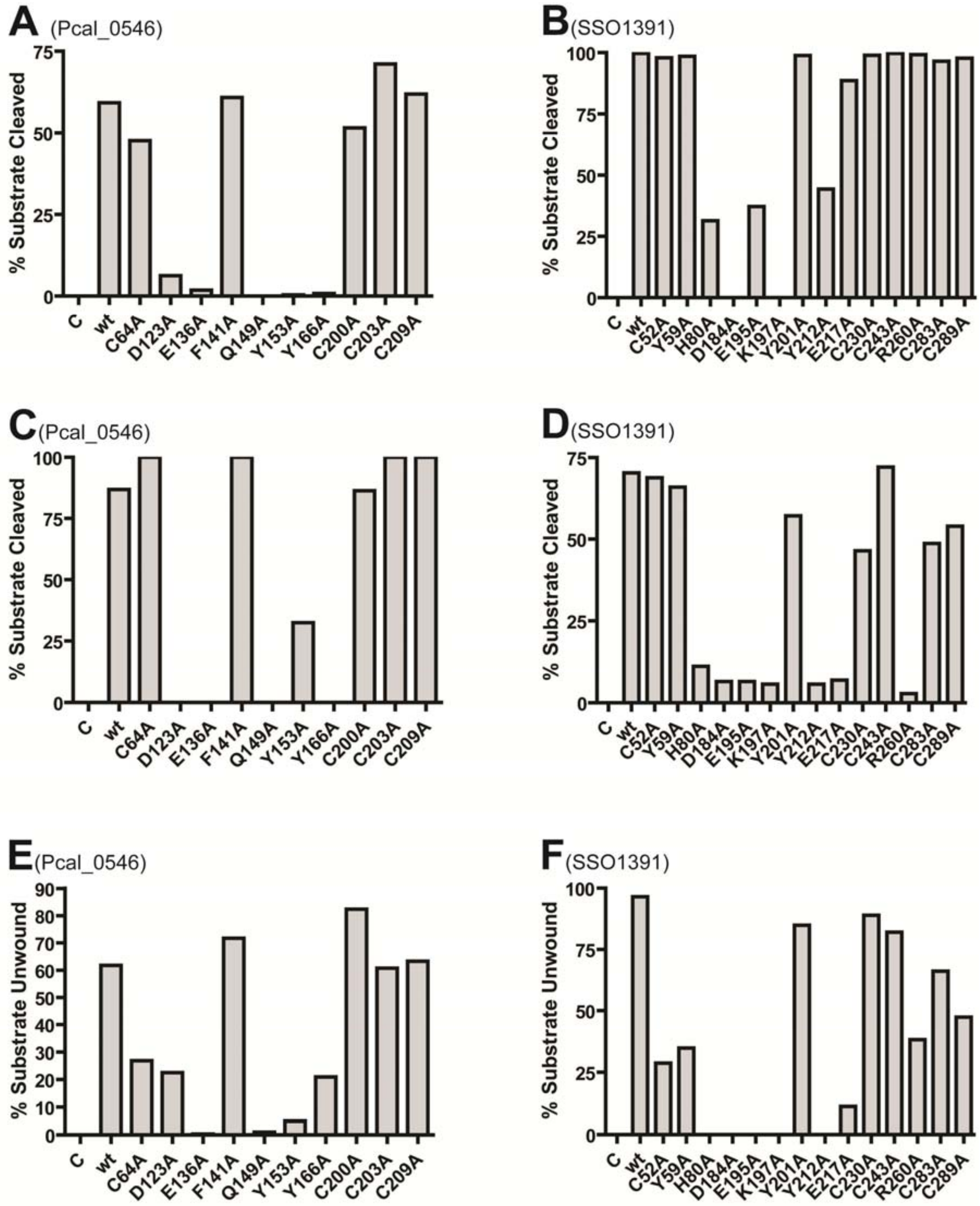

Supplementary Table S1. Oligonucleotides used in this work.

Substrate	Sequence	Length	Figures
DNA1	GTCCGGTTTATCCCTGCTGGCGCGGGAACTCCA	34 nt	2A, B; S3C-J
SA 21/21 (top strand)	ACGCTATTTTCTCACTCTAATGAGGATTATTTTACTTAGACT	42 nt	3A, B; 5B, E, F; S3O, P
SA 21/21 (bottom strand)	TCAGATTCATTTTATTAGGAGATTAGAGTGAGAAAATAGCGT	42 nt	3A, B; 5A, E, F; S3O, P
SA 38/21 (top strand)	AATGAAACGCTATTTTCTCACTCTAATGAGGATTATTTTACTTAGACTCTATTGCGTAA	59 nt	S3M, N
SA 38/21 (bottom strand)	AATGCGTTATCTCAGATTCATAAATAATCCTCATTAGAGTGAGAAAATAGCGTTTCATT	59 nt	S3M, N
10C	CCCCCCCCCC	10 nt	2E

Supplementary Table S2. Oligonucleotides used for the preparation of dsDNA substrates.

Substrate	Oligonucleotides used (Supplementary Table 1)	Double-stranded region (nt)	Single-stranded region (nt)	Figures
SA21	2 and 3	21	21	3A, B; S3O, P
SA38	4 and 5	38	21	S3M,N

Supplementary Table S3. Data collection and refinement statistics of Pcal_0546.

**Beamline**	**SBC-19ID**	**SBC-19BM**
Wavelength (Å)	0.98	1.74
Resolution range (Å)[b]	39.4- 2.65 (2.70 - 2.65)	40-2.80 (2.90 - 2.80)
Space group	I 4 2 2	I 4 2 2
Unit cell (a,b,c)	117.8,117.8, 89.4	117.8,117.8, 89.4
Total reflections	97592	34065
Unique reflections	9378	7997
Multiplicity [b]	10.4 (10.8)	4.3 (4.3)
Completeness (%) [b]	99.40 (100)	100 (99.7)
Mean I/sigma(I) [b]	40.6 (3.0)	29.0 (1.90)
Wilson B-factor	37.73	39.13
R-merge [b]	0.047(0.88)	0.047(0.91)
R-work [b]	0.228 (0.371)	
R-free	0.2534 (0.331)	
Number of non-hydrogen atoms	1738	
protein	1712	
ligands	5	
water	21	
Protein residues/waters/	215	
RMS (bonds)	0.010	

RMS (angles)	0.63	
Ramachandran favored/outliers (%)[c]	96.7/0	
Average B-factor: protein/ligands/water	48.5/38/32	

[a] $R_{\mathrm{merge}} = \Sigma_{hkl}\Sigma_i|Ii_{(}hkl_{)} - \langle I_{hkl}\rangle|/\Sigma_{hkl}\Sigma_i I_{i(hkl)}$, where $\mathrm{I}i_{(}hkl_{)}$ is the ith observation of reflection *hkl*, and $\langle I_{hkl}\rangle$ is the weighted average intensity for all observations *i* of reflection *hkl*.

[b] Numbers in parentheses are values for the highest-resolution bin.

[c] As defined by MOLPROBITY
